# Auditory-induced visual illusions in rodents measured by spontaneous behavioural response

**DOI:** 10.1038/s41598-019-55664-z

**Published:** 2019-12-16

**Authors:** Yuki Ito, Ryo Sato, Yuta Tamai, Shizuko Hiryu, Tomoko Uekita, Kohta I. Kobayasi

**Affiliations:** 1 Graduate School of Life and Medical Sciences, Doshisha University, 1-3 Tatara Miyakodani, Kyotanabe, 610-0394 Japan; 2 Department of Psychology, Kyoto Tachibana University, 34 Yamada-cho, Oyake, Yamashina-ku, Kyoto, 607-8175 Japan

**Keywords:** Perception, Sensory processing, Psychology

## Abstract

When two brief sounds are presented with a short flash of light, we often perceive that the flash blinks twice. This phenomenon, called the “sound-induced flash illusion”, has been investigated as an example of how humans finely integrate multisensory information, more specifically, the temporal content of perception. However, it is unclear whether nonhuman animals experience the illusion. Therefore, we investigated whether the Mongolian gerbil, a rodent with relatively good eyesight, experiences this illusion. The novel object recognition (NOR) paradigm was used to evaluate the gerbil’s natural (i.e., untrained) capacity for multimodal integration. A light-emitting diode embedded within an object presented time-varying visual stimuli (different flashing patterns). The animals were first familiarised with repetitive single flashes. Then, various sound stimuli were introduced during test trials. An increase in exploration suggested that the animals perceived a flashing pattern differently only when the contradicting sound (double beeps) was presented simultaneously with a single flash. This result shows that the gerbil may experience the sound-induced flash illusion and indicates for the first time that rodents may have the capacity to integrate temporal content of perception in a sophisticated manner as do humans.

## Introduction

When two sensory modalities receive conflicting information simultaneously, the perception in one modality is sometimes modified to align with the information in the other modality to construct a coherent multi-modal percept^1^. A prominent example, the “McGurk effect” (or “McGurk–MacDonald illusion”), demonstrates that listening to the sound /ba/ with a video clip showing a person’s lip uttering /ga/ often results in a combined auditory perception, such as “da”^2^. That audio-visual integration shows how significantly visual information (i.e., articulatory movement) contributes to auditory speech perception. More recently, Shams and colleagues reported the “sound-induced flash illusion”, which demonstrates that the opposite interaction (i.e., an auditory modality altering a visual perception) can also occur. In the illusion, a brief flash accompanied by two brief sounds is often perceived as two flashes^3,4^. Because the sound-induced flash illusion is not related to human-specific perception (i.e., speech perception), unlike the McGurk effect, it is reasonable to assume that this type of multimodal integration is fairly common in many animal species. However, as far as we know, there is no experimental evidence that animals other than humans are capable of experiencing this illusion. The lack of an appropriate animal model has hindered our understanding of this integration at the cellular and network levels.

Here, we used the Mongolian gerbil, *Meriones unguiculatus*, as a subject because it has sensitive low-frequency hearing comparable to humans^5^ and is considered a standard laboratory rodent, particularly in auditory neuroscience^6–8^. The gerbil’s retina also has a well-developed cone system^9,10^, and behavioural measurements of the animal’s grating acuity have suggested that the visual system of the gerbil is well adapted to a diurnal lifestyle^11^. The circadian rhythms of their activities under natural light conditions also indicate that gerbils are not fully nocturnal^12^, and they show a greater diurnal tendency than domestic mice or laboratory rats^13^. Therefore, these behavioural and physiological features make the gerbil a practical and valuable animal model of auditory–visual integration in non-human animals.

In this study, we used the novel object recognition (NOR) paradigm to measure the gerbil’s perception. Many rodents, including rats^14^, mice^15,16^, and degus^17^, approach a novel object more frequently and spend more time exploring it than they do an object to which they have previously been exposed^18^. The NOR paradigm relies on the animal’s innate preference for novelty. Therefore, no prior training, such as operant conditioning, is required, which means potentially high throughput. More importantly, the paradigm is suitable for evaluating an animal’s natural (untrained) cognitive potential acquired through normal development. The analogue of the NOR paradigm in human infants and non-human primates, the preferential looking time paradigm, reveals subjects’ natural cognitive functions (e.g., syntax learning^19^, voice–face pairing^20^, and cross-modal numerical matching^21,22^). In most of rodent studies investigating multimodal integration, the animals were trained to evaluate their perception^23,24^. Thus, their untrained sensory potential for a multisensory environment is relatively unknown. We used the NOR paradigm to investigate whether the Mongolian gerbil has the capacity to perceive the sound-induced flash illusion.

## Materials and Methods

### Subjects

In total, 43 Mongolian gerbils (27 males and 16 females), ranging in age from 1 to 4 months, were used in this study. All gerbils were bred and reared in our laboratory, and all were experimentally naïve. Each animal was housed with two to five other gerbils in a 20-cm width [W] × 40-cm length [L] × 17-cm height [H] cage with free access to food and water. The animal room was maintained on a 12-h light–dark schedule, and the temperature in the room was maintained at 22–23 °C with approximately 50% relative humidity. The gerbils were handled for at least 5 days before testing to reduce handling stress. All experimental procedures were performed in accordance with guidelines established by the Ethics Review Committee of Doshisha University, and the experimental protocols were approved by the Animal Experimental Committee of the university.

### Experimental conditions

**Figure 1:**
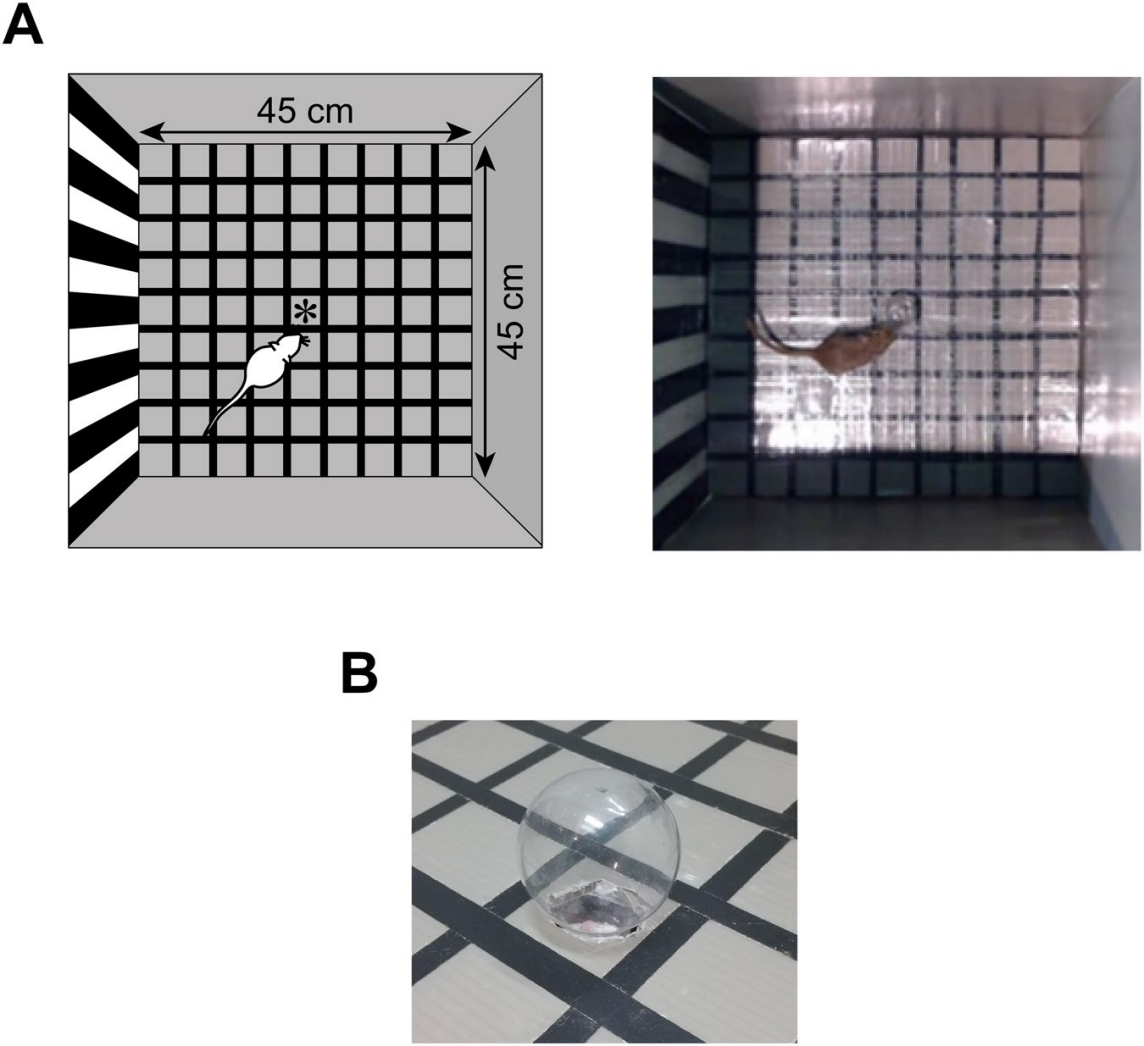
Experimental setting. (**A**) Schematised figure (left) and a picture (right) of the behavioural arena. (**B**) A picture of the object to be explored by the subject. The object was a glass bulb of 2.2-cm radius, and a white-light-emitting diode was embedded in the object.

All behavioural tests were conducted in a square behavioural arena (Fig. 1A; 45 [W] × 45 [L] × 55 cm [H]) located in a soundproof room. The brightness at floor level in the arena was 120 lx (measured with a lux meter; GL-03, be-s Co., Ltd., Tokyo, Japan). One wall of the arena was painted with vertical black and white stripes (3.5 cm [W]) as landmarks by which the gerbils could orient themselves. The floor was coloured with a square grid pattern (each square 5 × 5 cm^2^) marked with black tape (0.8 cm [W]). A bulb-shaped object (2.2-cm radius [R]) made of glass was placed at the centre of the arena to be explored (Fig. 1B). A white-light-emitting diode (LED) attached to a diffuser embedded within the object provided the visual stimulus. A loudspeaker (FT28D, Fostex, Tokyo, Japan) for presenting the auditory stimulus was set 100 cm above the arena. The amplitude of the sound stimulus was calibrated with a microphone (Type 1, ACO Pacific Inc., Aichi, Japan) placed at the centre of the arena at the height of the animal’s head (3 cm above the floor). The animal’s position during the experiment was monitored and recorded at 30 frames per second with a video camera (LifeCam HD-5000, Microsoft Inc., WA, USA) set next to the loudspeaker.

### Stimuli

**Figure 2:**
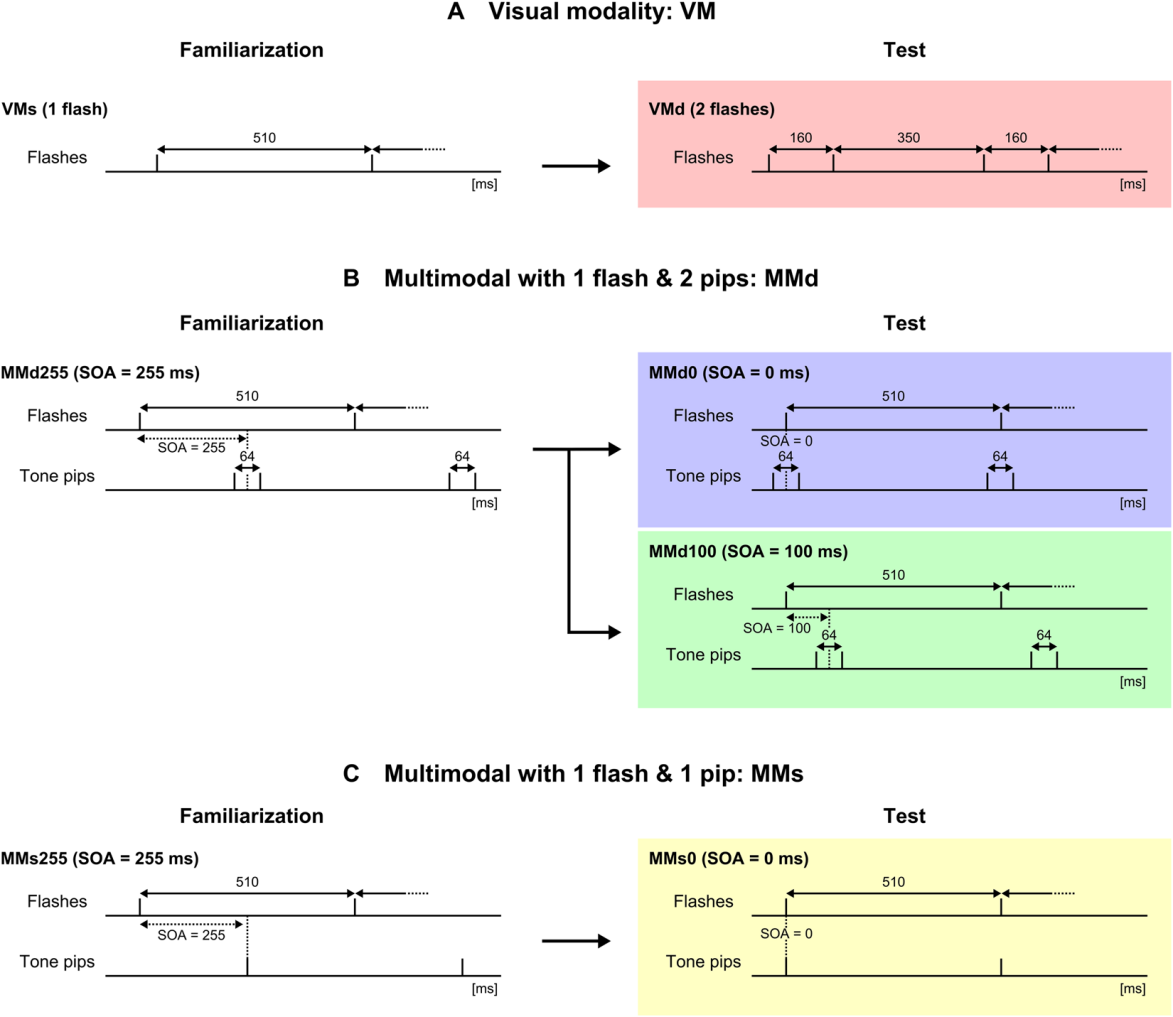
Temporal profile of each stimulus. The flash and tone pip were repeatedly presented during the trial. (**A**) Stimulus in the visual modality (VM) experiment. Only the visual stimulus was presented. A single flash (VMs) was presented during the familiarisation session, and a double flash (VMd) was presented during the test session. (**B**) The stimulus in the multimodal experiment using double tone pips (MMd). The visual stimulus was a single flash, and the auditory stimulus was double tone pips. Three types of stimulus onset asynchrony (SOA) were employed: 0 ms (MMd0), 100 ms (MMd100), and 255 ms (MMd255). (**C**) The stimulus in the multimodal experiment using a single tone pip (MMs). The visual stimulus was a single flash, and the auditory stimulus was a single tone pip. Two SOAs were employed: 0 ms (MMs0) and 255 ms (MMs255). The SOA was changed in the test session in both multimodal experiments (**B,C**).

The visual stimulus was a brief light (duration, 10 ms) presented by the LED inside the object. The intensity of the flash, measured 1 cm from the object, was 25 lx. The auditory stimulus was a tone pip (duration, 7 ms; frequency, 4 kHz; amplitude, 75 dB SPL). The visual and auditory stimuli were repeatedly and continuously presented during the familiarisation and test sessions. Seven different stimulus configurations were used (Fig. 2). In the visual modality (VM) experiment, only the visual stimulus (i.e., no auditory stimulus) was presented. A single flash was repeated (VMs) with an inter-onset interval (IOI) of 510 ms during the familiarisation sessions for the experiment. A double flash (VMd) was repeated at the same IOI during the test session. Each double flash consisted of paired single flashes with an IOI of 160 ms (Fig. 2A).

In the multimodal (MM) experiment, both the visual and auditory stimuli were presented, and the stimulus onset asynchrony (SOA) between them was changed for the test session. The IOI of the visual stimulus (flash) was 510 ms. Five stimulus configurations were used: MMd255, MMd0, MMd100, MMs255, and MMs0, where “d” and “s” represent double and single tone pips, respectively, and the number represents the SOA in ms. In the familiarisation session with double tone pips (MMd), a single flash and a double tone pip consisting of two tones separated by a 64-ms IOI were repeated with an SOA of 255 ms (MMd255; Fig. 2B). In the test trials, an SOA of 0 ms (MMd0) or 100 ms (MMd100) was tested. In the familiarisation session with a single tone pip (MMs), a single flash and a single tone pip were repeated with a 255-ms SOA (MMs255; Fig. 2C). In the test session, the SOA was 0 ms (MMs0). All visual and auditory stimuli were generated with customised programs (MATLAB; MathWorks, MA, USA).

### Behavioural procedure

Our experimental paradigm was a standard NOR procedure^25,26^. A habituation session was conducted first, followed by a familiarisation session, and then a test session. In the habituation session, each animal was allowed to explore the behavioural arena freely, with no flash-emitting object, for 30 min on 4 days. No visual or auditory stimulus was presented during these sessions. In the familiarisation session, the animals were habituated to the arena, and a flash-emitting object was set at the centre of the arena. Either the visual and auditory stimuli was presented (MM experiment) or only the visual stimulus (VM experiment). Each animal was released at the same position and allowed to explore the arena. Each trial lasted 5 min and was repeated five times, with a 2-min inter-trial interval. During the interval, the floor and object were cleaned with 80% ethanol to remove any odours, and the animal was isolated from its cage-mates in a cylinder-shaped box (12.4 [R] × 37.8 cm [H]). The same stimuli were presented during the familiarisation session. After the fifth familiarisation trial, the test session began following an identical 2-min interval. The test session lasted 5 min and was conducted only once.

### Analysis

Each animal’s exploration of the object was quantified as the duration of contact with the object. ‘Contact duration’ was defined as the total period during which the subject was touching the object with its snout or forepaw within the first 90 s of each trial; it was measured by counting the number of frames showing exploratory behaviour. Two experts, blinded to the experimental conditions, manually evaluated the behaviour, and their scores correlated significantly (R = 0.91, P < 0.01). Changes in contact duration (test minus fifth familiarisation trial) were tested if different from zero using t-test with a significance level of P < 0.05.

## Results

**Figure 3:**
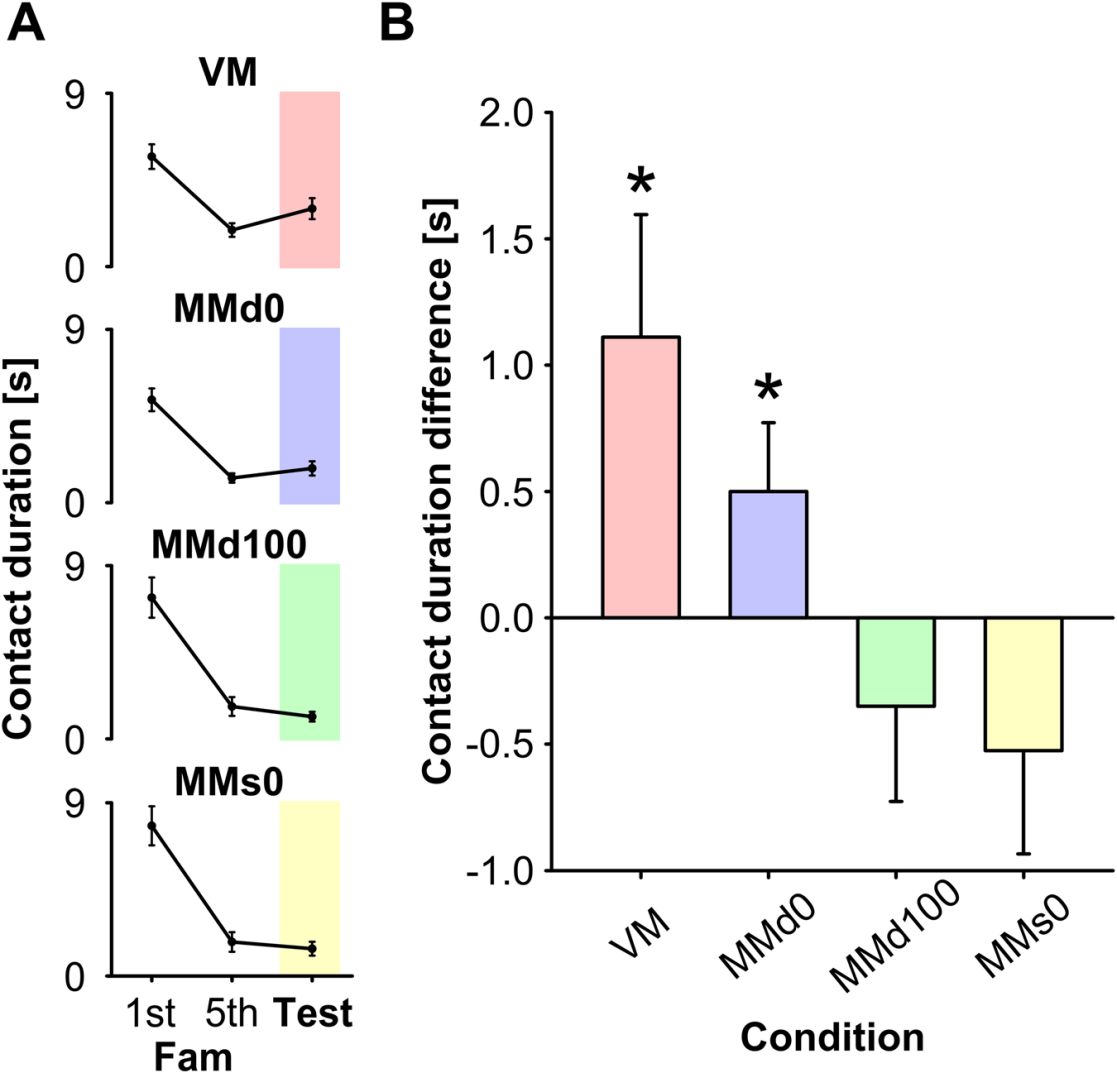
Effect of stimulus onset asynchrony (SOA) on the recognition of a flashing pattern. Error bars indicate standard errors. (**A**) The contact durations in the first and fifth (the last) familiarisation trial (Fam) and the test trial under all stimulus conditions. (**B**) Change in contact duration (test minus fifth familiarisation trial) with different SOA. Contact duration increased significantly in the test trial compared with that in the fifth familiarisation trial (255-ms SOA) when the animals were exposed to the 0-ms SOA (MMd0), but not when they were exposed to the 100-ms SOA (MMd100). In addition, a noncontradictory audio–visual stimulus pair (MMs0) did not produce a significant increase in the test trial compared with the fifth familiarisation trial. *P < 0.05.

Two gerbils experienced seizures during the habituation or experimental period, and were excluded from the analysis. In the VM experiment, the animals were repeatedly exposed to single flashes (VMs) during the familiarisation trial, and they explored the object less as the trial proceeded (Fig. 3A VM). After the number of flashes changed (VMd) in the test trial, their exploration of the object increased significantly compared with that in the fifth familiarisation trial (1.90 ± 0.35 vs. 3.01 ± 0.54 s, mean ± standard error of the mean; *t*_(8)_ = 2.29, P < 0.05; Fig. 3B VM). These results confirmed that the NOR paradigm is suitable for assessing the perception of a temporally changing visual stimulus.

In the MM experiments, the gerbils’ exploration of the object decreased as the trial progressed during the familiarisation sessions, as was observed in the VM experiments (MMd255: 6.24 ± 0.6 s vs. 1.46 ± 0.25 s; MMs255: 7.81 ± 1.01 s vs. 1.77 ± 0.51 s). The contact durations in the first and fifth familiarisation trials and the test trial under all conditions are shown in Fig. 3A. With test stimulus MMd0, in which a flash and double tone pips were presented with 0-ms SOA, the animals explored the object significantly longer than they had in the fifth familiarisation trial (1.28 ± 0.24 vs. 1.78 ± 0.37 s, *t*_(10)_ = 1.83, P < 0.05; Fig. 3B MMd0). With test stimulus MMd100, in which a flash and double tone pips were presented with 100-ms SOA, no significant increase in the exploratory period was observed (1.68 ± 0.49 vs. 1.16 ± 0.25 s, *t*_(8)_ = 1.29, P = 0.12; Fig. 3B MMd100). In the MMs experiments, in which a single flash and a single tone pip were presented, reducing the SOA from 255 ms (familiarisation trial) to 0 ms (test trial), the exploratory period did not increase (1.77 ± 0.51 vs. 1.42 ± 0.36 s, *t*_(11)_ = 0.93, P = 0.19; Fig. 3B MMs0).

## Discussion

The results of the VM tests showed that changing the number of flashes triggered a significant increase in the gerbils’ exploratory behaviour. Previous studies using the NOR paradigm have demonstrated that animals are more interested in an object and explore it more thoroughly when the object’s properties (shape, size and colour) and/or position are altered (for review see Ennaceur^27^). Recent studies that combined a Y-shaped apparatus with a NOR task demonstrated that rats visually recognise an object based on their perception of its features with tactile senses^28,29^, demonstrating that this task can be used to test multimodal recognition. Because we wanted to use the NOR paradigm to evaluate the perceptual content of a temporally dynamic visual signal, particularly its multimodal effect, the pattern of flashes emitted by an object was altered as the experimental variable. As observed in the typical NOR paradigm, the gerbils showed decreasing contact with the object during familiarisation, but when the number of flashes was altered from one to two, the gerbils explored the object for a longer period (Fig. 3A VM). This increase in exploration suggests that the gerbils recognised the change in the temporal pattern of flashing, and the difference was sufficient to solicit exploratory behaviour toward the object. This result demonstrated that the NOR paradigm can be used to assess this animal’s visual temporal perception and recognition.

In humans, when two beeps are presented together with a single flash, the flash is often perceived twice^3,4^. Whereas, as the amount of SOA between the auditory and visual stimuli increases, the illusory perception occurs less frequent^4^. A study by Bidelman (2016) systematically analysed the effect of SOA in musicians and non-musicians and demonstrated that the temporal window of sensory integration was <100 ms in musicians and ~200 ms in non-musician subjects^30^. Our results demonstrate that exploration of the stimulus object increased significantly under the MMd0 condition, but not under the MMd100 condition (Fig. 3B). We interpret these data as indicating that the animals perceived the MMd255 stimulus as a single flash during the familiarisation session, but experienced the MMd0 stimulus as a double flash (an illusory flash), as humans do. However, they did not experience the MMd100 stimulus as a double flash (or did so to a much smaller extent). These results support the idea that animals perceive the sound-induced flash illusion and that the temporal window for audio–visual integration is similar to that in humans.

The contact duration in MMd0 (Fig. 3B) is almost half that in VM (while the difference was not statistically significant (*t*_(18)_ = 1.15, P = 0.26)). Many human studies have shown that the illusory perception (double flash illusion) does not always occur. For example, Shams *et al*. reported that the illusion occurred in ca. 70% of tests, even under the best SOA conditions^4^. Other research by Andersen and colleges (2004) reported that subjects perceived two flashes in 55% of all trials^31^. Recent research confirmed that the illusory flash was perceived only in ca. 60% of events^32^. Therefore, even if gerbils experience the illusory flashes as often as humans, they still perceive MMd0 as double flashes in 55–70% of all presentations, and the probabilistic nature of the illusion could explain the response difference between VM and MMd0. The greater increase from a familiarised stimulus (i.e., single flash) in VM could promote stronger exploratory behaviour than in MMd0.

One might argue that the data only suggest that the animals perceived flashes in MMd0 as different from those in MMd255, not necessarily as a double flash. For example, a simultaneous auditory stimulus might somehow enhance the saliency of the visual stimulus enough to trigger exploration^33^. Because those multimodal enhancements are reported to be the most prominent at the 0-ms SOA, we introduced the MMs0 (0-ms SOA) as a control. The results under the MMs condition demonstrate that changing the SOA from 255 to 0 ms was insufficient to produce a significant behavioural response (Fig. 3B MMs0), suggesting the showing that stimulus simultaneity alone was insufficient to change exploratory behaviour. The incongruence of the two sensory cues (i.e., single or double) and the stimulus timing are important to affect the behaviour, and the requirements are comparable to those observed during a sound-induced flash illusion in human research^34^. One might also argue that a double tone pip alone is sufficient to induce exploration, and a flash presented with a certain delay somehow cancels the effect. Our additional analysis suggests that this is rather unlikely. Specifically, the contact duration in the first familiarisation trial in VMs (single flash without double tone pips: Fig. 3A VM first) was not statistically significantly different from the first familiarisation in MMd255 (single flash with double tone pips: Fig. 3A MMd0 first and MMd100 first; *t*_(27)_ = 0.53, P = 0.60). Whereas this analysis on MMd cannot be a complete substitute for testing on additional control stimulus (i.e., unimodal double-tone-pip), the statistical result suggests that the double tone pips did not significantly increase exploration. Overall, our data should not be taken as direct evidence that animals perceive the auditory-induced illusory flash, and our results require further behavioural research, such as establishing whether the animals can discriminate the physically presented double flash from the illusory one^35^.

The neural mechanism of sensory integration has been the focus of many studies. Rodents integrate multisensory input both behaviourally and physiologically^36–38^. However, as far as we know, there is no behavioural evidence showing that rodents experience multisensory illusions, except the study by Wada *et al*. on a visual–tactile illusion^23^. Our results present the possibility that the visual recognition of untrained gerbils was altered by the auditory stimulus, suggesting that the species have the capacity to integrate temporal content of perception in a sophisticated manner as do humans, and also that the species is a good animal model for investigating the auditory-induced flash illusion. Several human studies have already investigated the brain regions involved in this illusion. Using functional magnetic resonance imaging, Zhang and Chen (2006) showed that elevated activity in the visual cortex is associated with this illusory perception^39^. Mishra *et al*. (2007) provided evidence that rapid input from the auditory and multisensory areas modifies the activity of the visual cortex and promotes the illusion^40^. Because neither of these regions nor the proposed mechanism is specific to humans, these systems could serve as the neural basis for the integration in rodent and as well. Future research in rodents may provide cellular-level insight into the sound-induced flash illusion.
